# Analysis of fatal cases of severe fever with thrombocytopenia syndrome in Jiangsu province, China, between 2011 and 2022: A retrospective study

**DOI:** 10.3389/fpubh.2023.1076226

**Published:** 2023-03-23

**Authors:** Shuyi Liang, Wei Xie, Zhifeng Li, Nan Zhang, Xiaochen Wang, Yuanfang Qin, Changjun Bao, Jianli Hu

**Affiliations:** ^1^Jiangsu Provincial Center for Disease Control and Prevention, Acute Infectious Disease Control and Prevention Institute, Nanjing, China; ^2^Jiangsu Provincial Center for Disease Control and Prevention, Institute of Food Safety and Assessment, Nanjing, China

**Keywords:** SFTS, fatality, Cox regression, risk factors, retrospective study

## Abstract

**Introduction:**

Severe fever with thrombocytopenia syndrome (SFTS) is an emerging infectious disease caused by the SFTS virus (SFTSV), which has a high fatality rate. This disease has become increasingly prevalent in recent years in Jiangsu province, with a noticeable rise in its incidence. Notably, fatal cases have also been increasing. Our study aimed to analyze the epidemiological characteristics and risk factors associated with the fatal cases of SFTS in Jiangsu province from 2011 to September 2022.

**Methods:**

A retrospective study was performed among 698 SFTS cases during 2011-2022 in Jiangsu Province, China. Cox regression analyses were used to determine the dependent and independent risk factors that affected patient survival time. ArcGIS 10.7 was used for the visualization of the geographical distribution of the deaths from SFTS.

**Results:**

There were 698 SFTS cases reported, with an increasing incidence, over the 12-year period. Among these cases, 43 deaths were reported. Fatal cases of SFTS were reported in 12 district counties from 2011 to 2022. Notably, most of the deaths occurred in Lishui county of Nanjing City. The median age of those who died was 69 years, with age ranges from 50 to 83 years. Multivariable Cox regression analysis showed that older age (>70) and living in Lishui county were risk factors for death from SFTS in Jiangsu province. Therefore, older adults aged over 70 years and residing in Lishui county were the high-risk group for SFTS mortality.

**Discussion:**

Over the past 12 years, we have observed a consistent rise in the incidence of SFTS, accompanied by a relatively high case fatality rate, making it a critical public health issue. Therefore, it is urgently necessary to study the impact of meteorological factors on SFTS epidemics and devise prevention and control strategies.

## Introduction

Severe fever with thrombocytopenia syndrome (SFTS) is an emerging infectious disease caused by the SFTS virus (SFTSV). It was first discovered in China in 2009 (1). The vast majority of SFTS cases were identified in Shandong, Hubei, Henan Anhui, Liaoning, Zhejiang, and Jiangsu provinces. Notably, over the last decade in China, the number of SFTS cases has not only continued to increase but has also expanded its geographical distribution. In recent years, more than 15 provinces in China have reported SFTS cases (2). Furthermore, SFTS cases have also been identified in other countries, such as Japan, South Korea, Vietnam, the United Arab Emirates, and the United States (3–7).

The main clinical manifestations of SFTS included high fever, leukopenia, thrombocytopenia, and multiple organ damage (8), with a mortality rate of more than 10% (9, 10). SFTSV is mainly transmitted through tick bites. Furthermore, SFTSV can also spread from person to person through direct contact with blood (11). Additionally, cases of SFTSV transmission from dogs and cats have also been reported and are considered a public health issue (12, 13). Owing to its life-threatening impact on public health, SFTS was chosen as one of the nine emerging diseases prioritized for research and development by the World Health Organization in 2017 (14).

In recent years, there has been a noticeable increase in the SFTS cases in Jiangsu province, with a corresponding rise in fatal cases. Given the severity of the situation, we conducted a retrospective analysis of the epidemiological data of fatal SFTS cases from 2011 to 2022. Through this analysis, we aimed to gain more specific knowledge about the epidemiological characteristics and risk factors of death in patients with SFTS and wanted to focus on the high-risk groups.

## Materials and methods

### Study area

Jiangsu is a coastal province located in the middle of the east coast of China (30°45′-35°08′ N, 116°21′-121°56′ E). This region experiences a monsoon climate that shifts from subtropical to temperate (Figure 2) and features a landscape of plains, mountains, and hills. The county or district is the study unit. Jiangsu province is administratively divided into 95 counties or districts.

### Case definition

One patient had a fever (≥38.0°C) along with other symptoms (e.g., digestive symptoms and bleeding) and risk factors related to epidemiology (such as tick exposure 2 weeks before illness onset or working as a farmer), and clinical testing data showed thrombocytopenia and leukocytopenia. A suspected patient was classified as a confirmed case if one or more of the following conditions were met: (1) SFTSV RNA was identified, (2) seroconversion or a 4-fold increase in antibody titers between paired serum samples were collected at intervals of 2 weeks or more, and (3) the SFTSV was isolated from cell culture (15).

### Data collection

The data on SFTS cases between January 2010 and September 2022 in Jiangsu province were obtained from the Nationwide Notifiable Infectious Diseases Reporting Information System (NIDRIS). Every case was identified based on a laboratory or clinical diagnosis. The data on SFTS cases included information on the patient's address, age, gender, occupation, and date of onset. In addition, the demographic information was obtained from the Jiangsu statistical yearbook.

### Statistical analysis

SPSS version 25.0 software (IBM, Armonk, NY, USA) was used to perform data analysis. Cox regression analyses were used to determine the dependent and independent risk factors that affected patient survival time. ArcGIS 10.7 (ESRI, Redlands, CA, USA) was used for the visualization of the geographical distribution of the deaths from SFTS.

### Ethics statement

This present study was authorized by the ethics committee of the Jiangsu Provincial CDC.

## Results

### Epidemiological features

**Figure 1:**
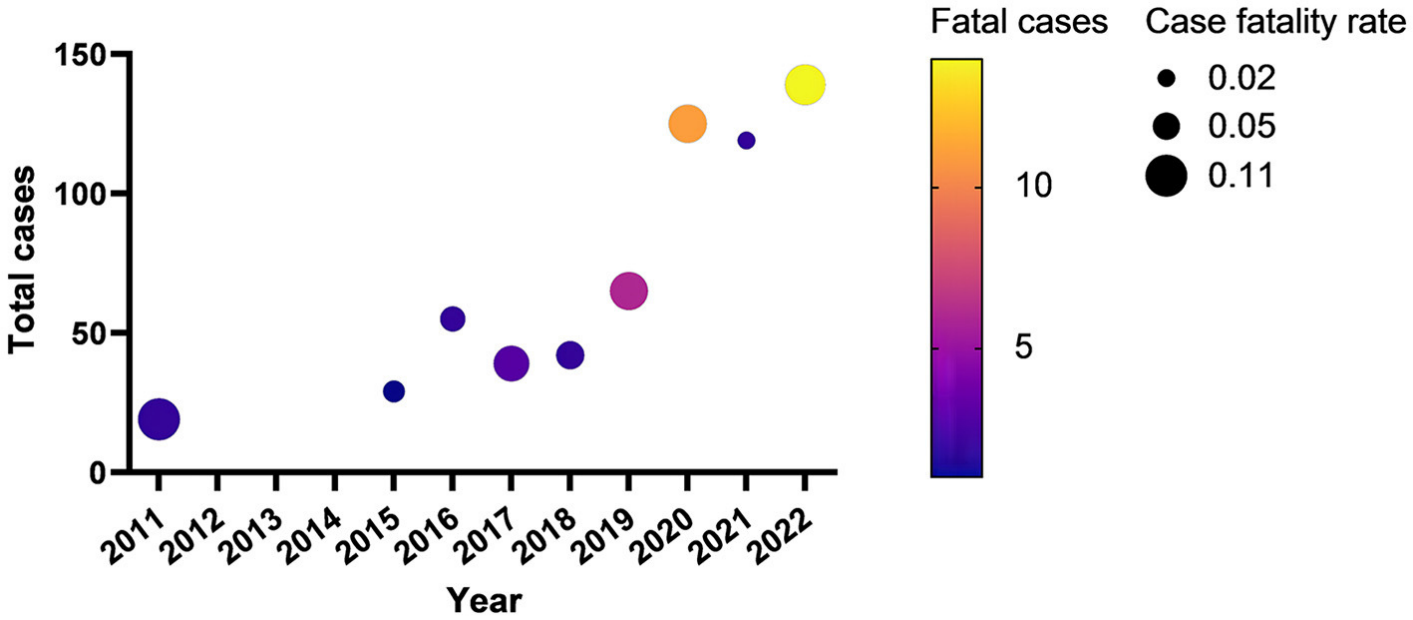
The fatal cases and the case fatality rate from 2011 to 2022.

**Figure 2:**
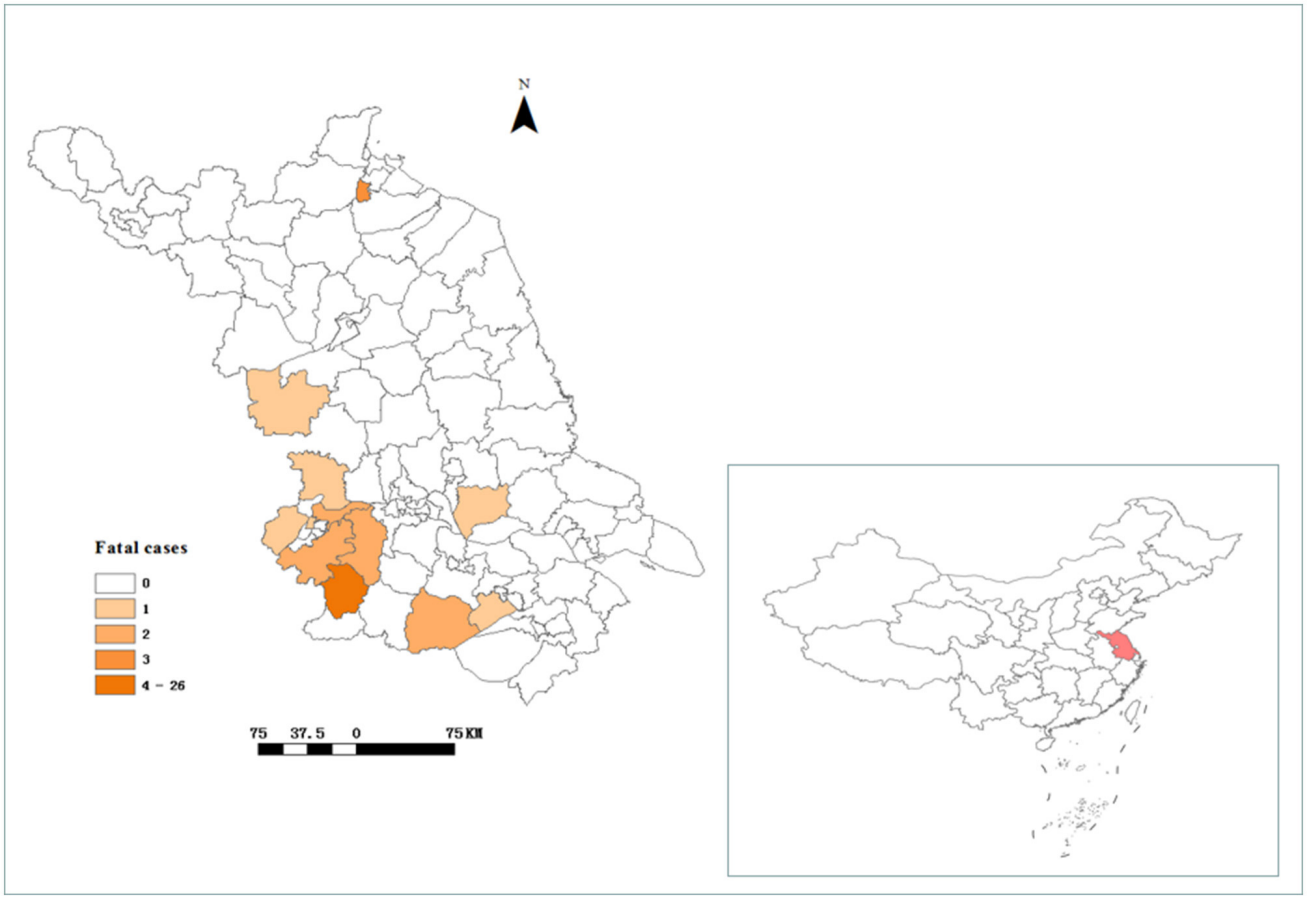
The location of Jiangsu province and the geographical distribution of fatal cases of SFTS in Jiangsu province from 2011 to 2022.

From 2011 to September 2022, 698 confirmed SFTS cases were identified in Jiangsu province, with an increasing trend. Among the 698 confirmed cases, 43 fatal cases were reported. The annual fatal cases ranged from the lowest (0) in 2012–2014 to the highest (14) in 2022. The fatality rate was up to over 10% in 2011 and 2022 (Figure 1). The fatal cases in Jiangsu province were distributed among 12 district counties from 2011 to 2022 (Figure 2). Notably, the majority of the deaths occurred in Lishui county of Nanjing City, with a fatality rate of 17.2% (23/134).

**Figure 3:**
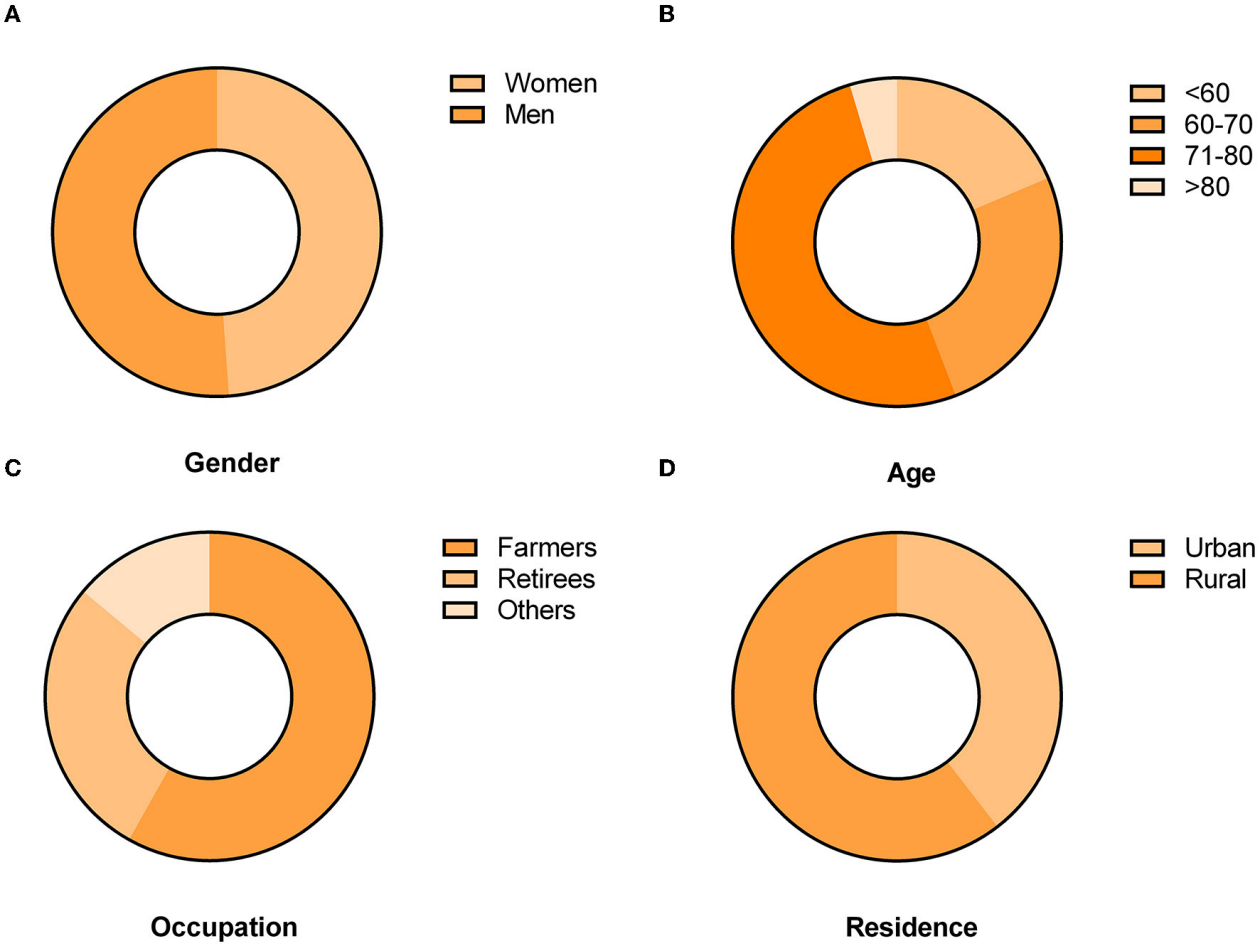
Gender, age group, occupation, and residence distributions of the number of fatal cases of SFTS in Jiangsu province, 2011–2022. **(A)** Gender distribution of fatal SFTS cases. **(B)** Age group distribution of fatal SFTS cases. **(C)** Occupation distribution of fatal SFTS cases. **(D)** Residence distribution of fatal SFTS cases.

Of the 43 deaths, 22 (51.2%) of them were men, with a male-to-female ratio of 1.05:1. The median age at death was 69 years, ranging from 50 to 83 years. Furthermore, most of the people who died were over 60 years. Additionally, the majority of the deaths occurred in rural areas. Among the deaths, 58.1% were farmers, 27.9% were retirees, and the rest were others (Figure 3).

**Figure 4:**
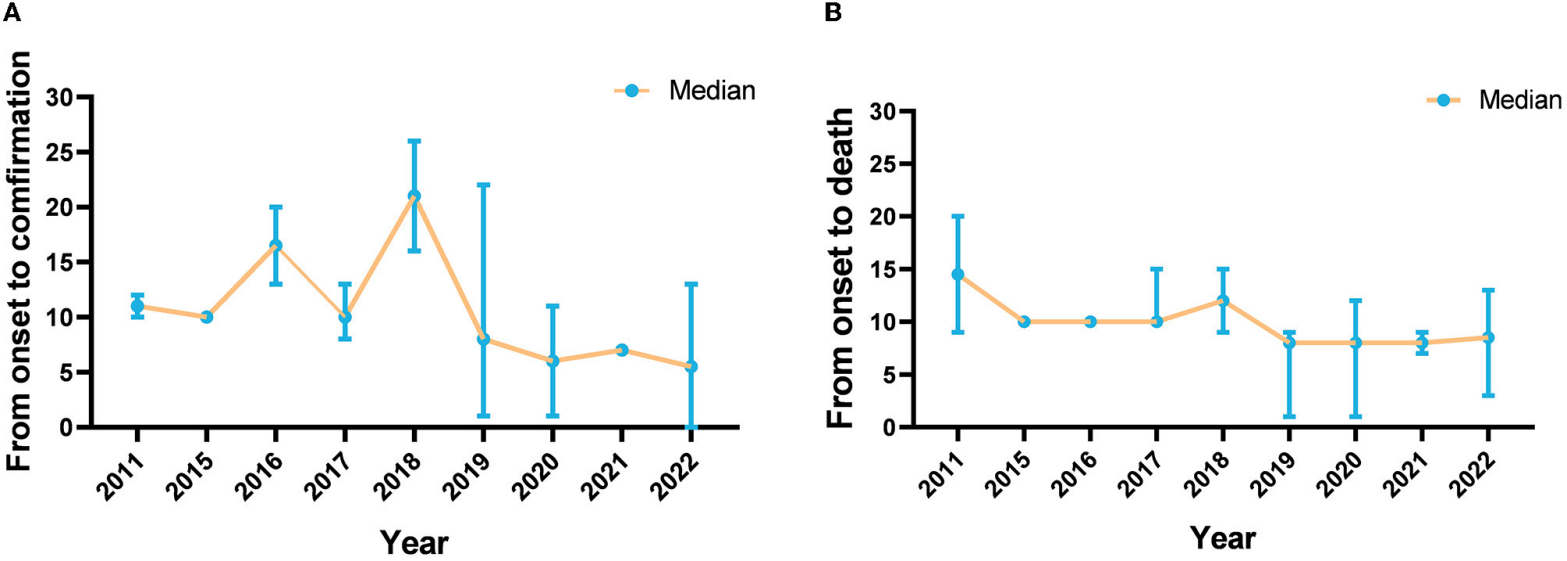
The interval between illness onset, confirmation, and death of SFTS cases in Jiangsu province from 2011 to 2022. **(A)** Is the interval between illness onset and confirmation, and **(B)** is the interval between illness onset and death.

The median time from illness onset to diagnosis in the 43 fatal cases was 8 days, and the annual median time from illness onset to diagnosis in cases from 2011 to 2022 ranged from 5.5 to 21 days, with medians of 11, 10, 16.5, 10, 21, 8, 6, 7, and 5.5 days in each respective year. Moreover, the median time from illness onset to death in the 43 fatal cases was 9 days. The annual median time from illness onset to death in cases from 2011 to 2022 ranged from 8 to 14.5 days, with medians of 14.5, 10, 10, 10, 12, 8, 8, 8, and 8.5 days in each respective year (Figure 4).

### Risk factors for death

We performed Cox regression analyses on several indicators, including gender, age, occupation, and district. Univariable Cox regression analysis revealed that older age (>70) and living in Lishui county were risk factors for fatal outcomes (Table 1). We further included two significant variables in our multivariable Cox regression analysis, which showed that older age (>70) and living in Lishui county were independent risk factors for death. The complete results of the multivariable analysis are shown in Table 2.

**Table 1:** Univariable Cox regression analyses of characteristics related to death in patients with SFTS.

** 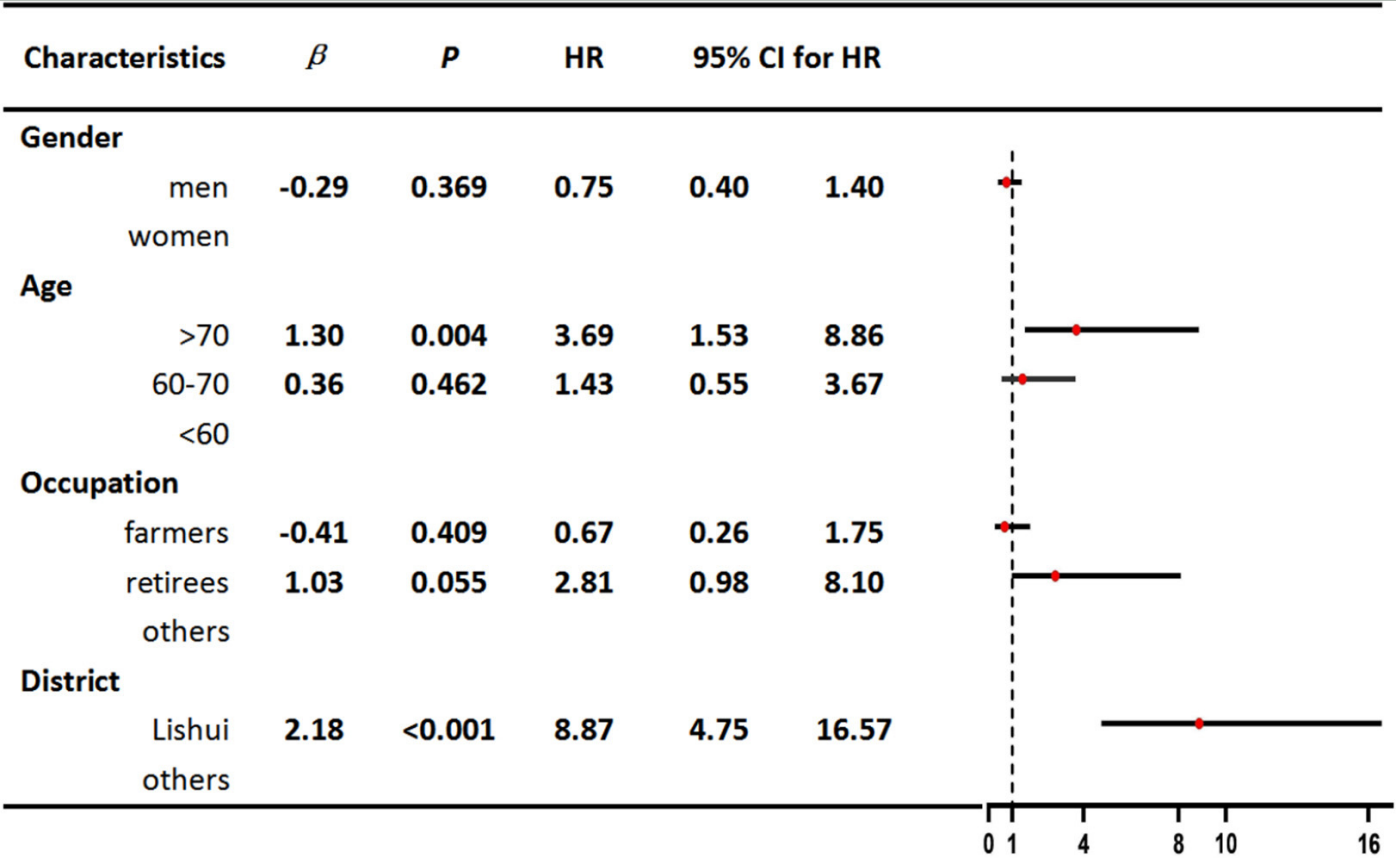 **

**Table 2:** Multivariable Cox regression analyses of characteristics related to death in patients with SFTS.

** 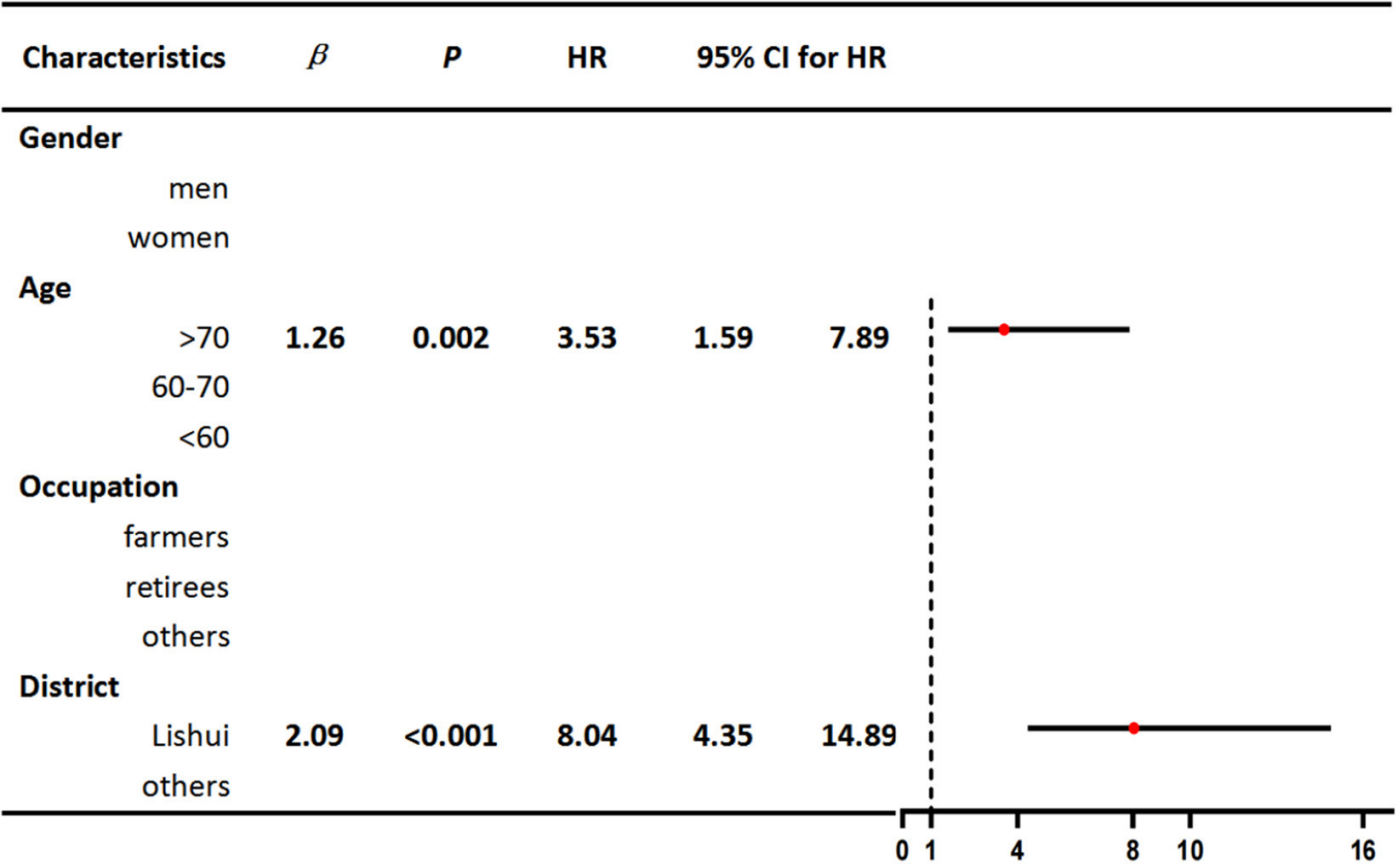 **

## Discussion

In the present study, we discovered that the annual incidence of SFTS has been increasing over the past 12 years, and the epidemic area has also expanded. The overall case fatality rate was 6.1% in Jiangsu province from 2011 to 2022. The results were slightly lower than those reported in other studies (16–21). The majority of SFTS fatal cases reported in Jiangsu province mostly occurred in the western and southern hilly regions. The areas included Lishui county, Haizhou county, and Yixing county. Unexpectedly, there were a large number of confirmed cases but few deaths in Xuyi county. The majority of deaths occurred in Lishui county, which had the highest fatality rate (17.2%). Numerous factors might have contributed to the results. First, there were abundant forest resources and the dominant tick species, *H. longicornis* (22, 23), in Lishui county. In addition, the local meteorological environment may have been conducive to the spread of the virus. Therefore, a thorough study of the impact of meteorological factors on SFTSV transmission in Lishui county is needed.

Most of the people who died were farmers residing in rural regions with a mountainous and hilly terrain. An age range of 71–80 years accounted for more than half the deaths. The results were in accordance with the previous studies (24, 25). The number of deaths related to men and women was similar. This result differs from that published in previous studies (26).

We also found that, among the 43 fatal cases, the median time between the onset of symptoms and confirmation of SFTS was 8 days, with a decrease from 1 day in 2011 to 5.5 days in 2022. In the early days of the SFTS epidemic, the median time was longer, which was due to the doctors' lack of extensive relevant clinical experience with SFTS. Notably, the median interval between the onset of symptoms and death was 9 days. The results showed that the interval was very small from the confirmation of SFTS to death. Delays and mistakes in the diagnosis might influence the prognosis of patients with SFTS (27, 28). To shorten the time between disease onset and its confirmation and to minimize the risk of fatalities, it is essential to enhance the capacity for diagnosing and treating SFTS in hospitals.

To control for potential confounding factors and estimate the direct effects of several possible causal risk factors, we applied a multivariate Cox regression model in our study. The results revealed that individuals over the age of 70 residing in Lishui county were at a greater risk of death from SFTS. On the one hand, elderly people infected with SFTSV might experience severe clinical symptoms that are related to a decline in immune function and comorbidities. On the other hand, we speculated that the main reason for the high fatality rate in Lishui county was the high rate of virus-carrying ticks in the local area. In the future, we will conduct further special investigations and research to verify this speculation. It is worth noting that Lishui county, as an area with extensive natural features, not only had a large number of confirmed cases but also a high fatality rate. Therefore, it was necessary to conduct further research on the impact of meteorological factors on the natural transmission cycle in the area of these natural features. Surprisingly, farmers were not found to be at high risk of death among patients with SFTS. The results may be related to the physical resilience and stamina of farmers.

However, this study had a few limitations. First, the data obtained from NIDRIS may have been underestimated because of underreporting, which includes cases where individuals did not seek medical attention and those who died before reaching a medical facility. Second, potential correlations between clinical indicators and outcomes were not investigated. Finally, this study could have been subject to confounding bias due to a lack of precise population control.

To date, we have paid less attention to the epidemiological characteristics of fatal SFTS cases. The deaths caused by SFTS in Jiangsu province have not yet been systematically and comprehensively analyzed. Therefore, our study provides the first comprehensive analysis of the epidemiological characteristics and risk factors associated with fatal SFTS cases in Jiangsu province. We observed a steady increase in incidence and a relatively high fatality rate in the past. Older adults over the age of 70 years residing in Lishui county were found to be the most vulnerable group for SFTS in Jiangsu province. These findings have significant implications for the precise control and prevention of SFTS. In the future, an in-depth study is urgently needed to elucidate the role of meteorology in the natural transmission cycle of the disease.

## Data availability statement

The original contributions presented in the study are included in the article/supplementary material, further inquiries can be directed to the corresponding authors.

## Ethics statement

This study was authorized by the Ethics Committee of Jiangsu Provincial CDC.

## Author contributions

SL: data curation, data analysis, manuscript writing, software, and visualization. WX: manuscript reviewing and editing. ZL, NZ, XW, and YQ: data curation and supervision. CB and JH: manuscript design. All authors have read and approved the final version of the manuscript.
